# Supplementary material for: Genomic characterisation of Eμ-Myc mouse lymphomas identifies Bcor as a Myc co-operative tumour-suppressor gene
Source: Nat Commun. 2017 Mar 6;8:14581. doi: 10.1038/ncomms14581 (PMC5343491; doi:10.1038/ncomms14581)
Supplement: Supplementary Information — Supplementary Figures, Supplementary Tables. [file ncomms14581-s1.pdf]

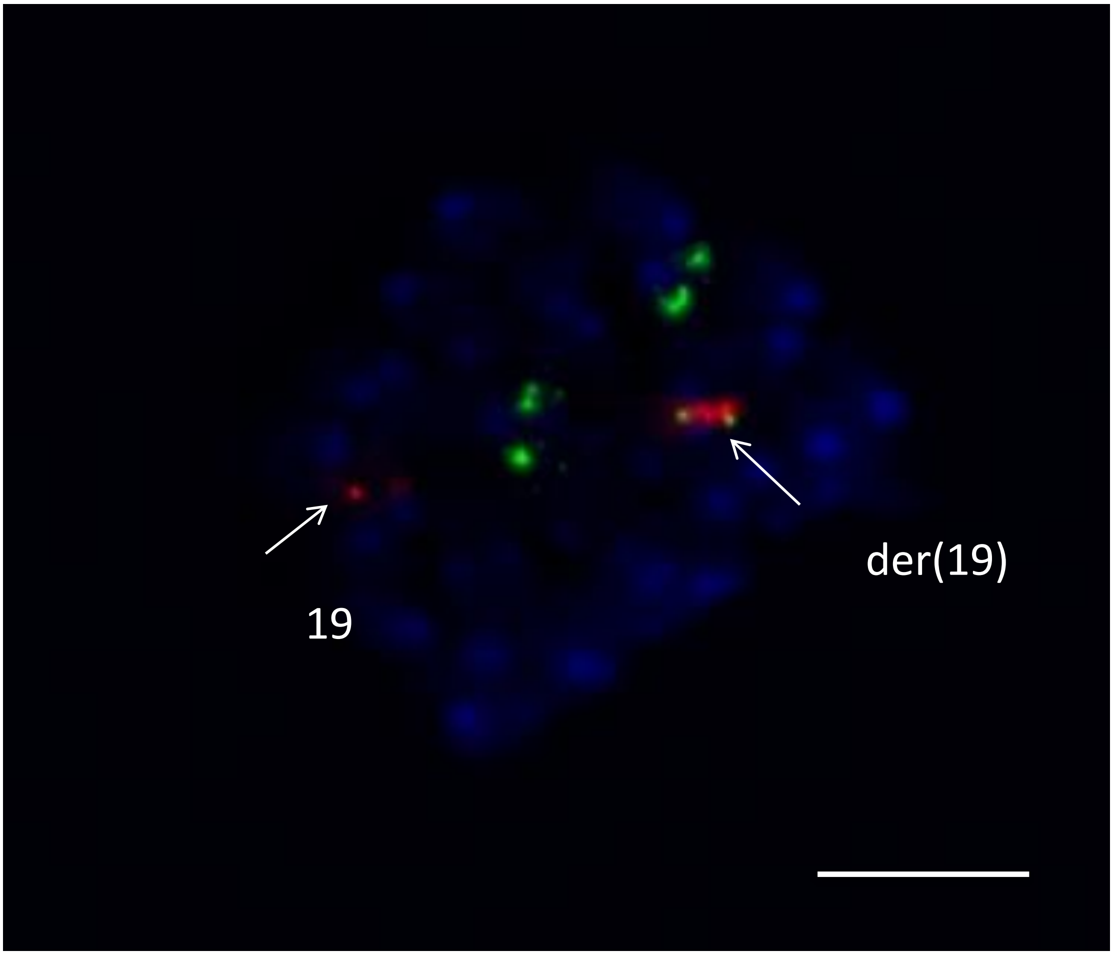

Supplementary Figure 1. BAC metaphase FISH on E***μ***-*Myc* transgenic cultured bone marrow cells. BAC RP23-307D14 (spectrum green) spans the MYC locus on chromosome 15. BAC RP23-324L2 (spectrum orange) spans JAK2 on chromosome 19. There is an extra, half strength green signal (MYC) co-localising with a red signal (Jak2) on the derivative chromosome 19 confirming that the transgene is located on chromosome 19. The red signal on the derivative 19 is always larger than the signal on the intact chromosome 19 which would support the presence of copy number gain in the region spanned by RP-324L2 but copy number cannot be accurately quantified. Scale bar = 10µm.

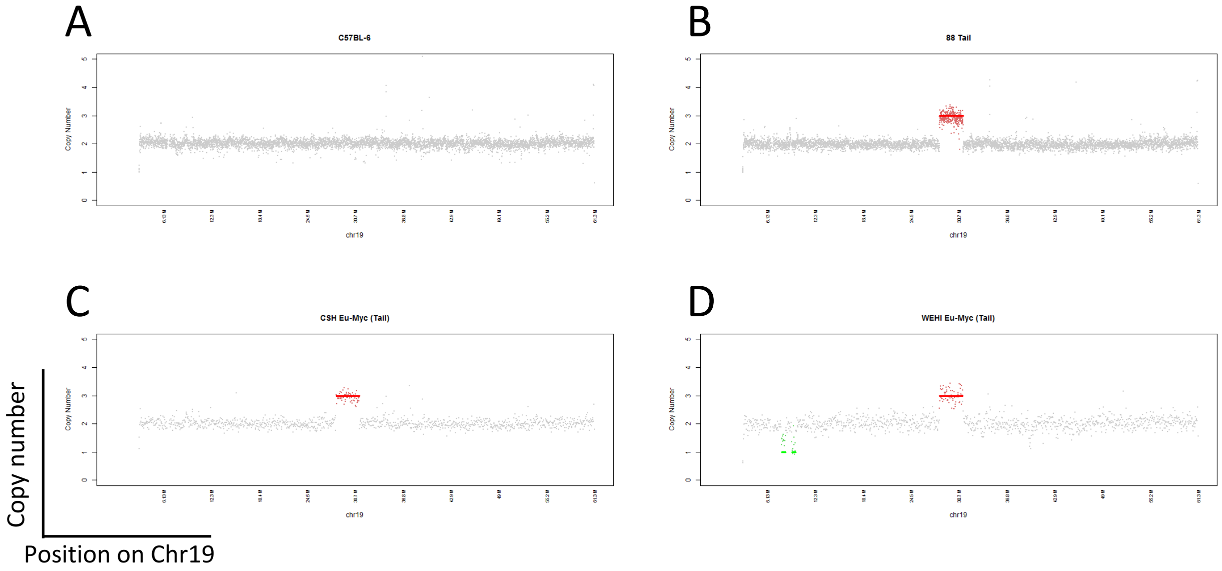

Supplementary Figure 2. Whole genome sequencing copy-number analysis highlighting germline amplification of the Chr19 segment proximal to E***μ***-*Myc* transgene insertion. A) C57BL/6 WT control B) E*μ*-*Myc* Peter MacCallum Cancer C) E*μ*-*Myc* Cold Spring Harbor D) E*μ*-*Myc* WEHI.

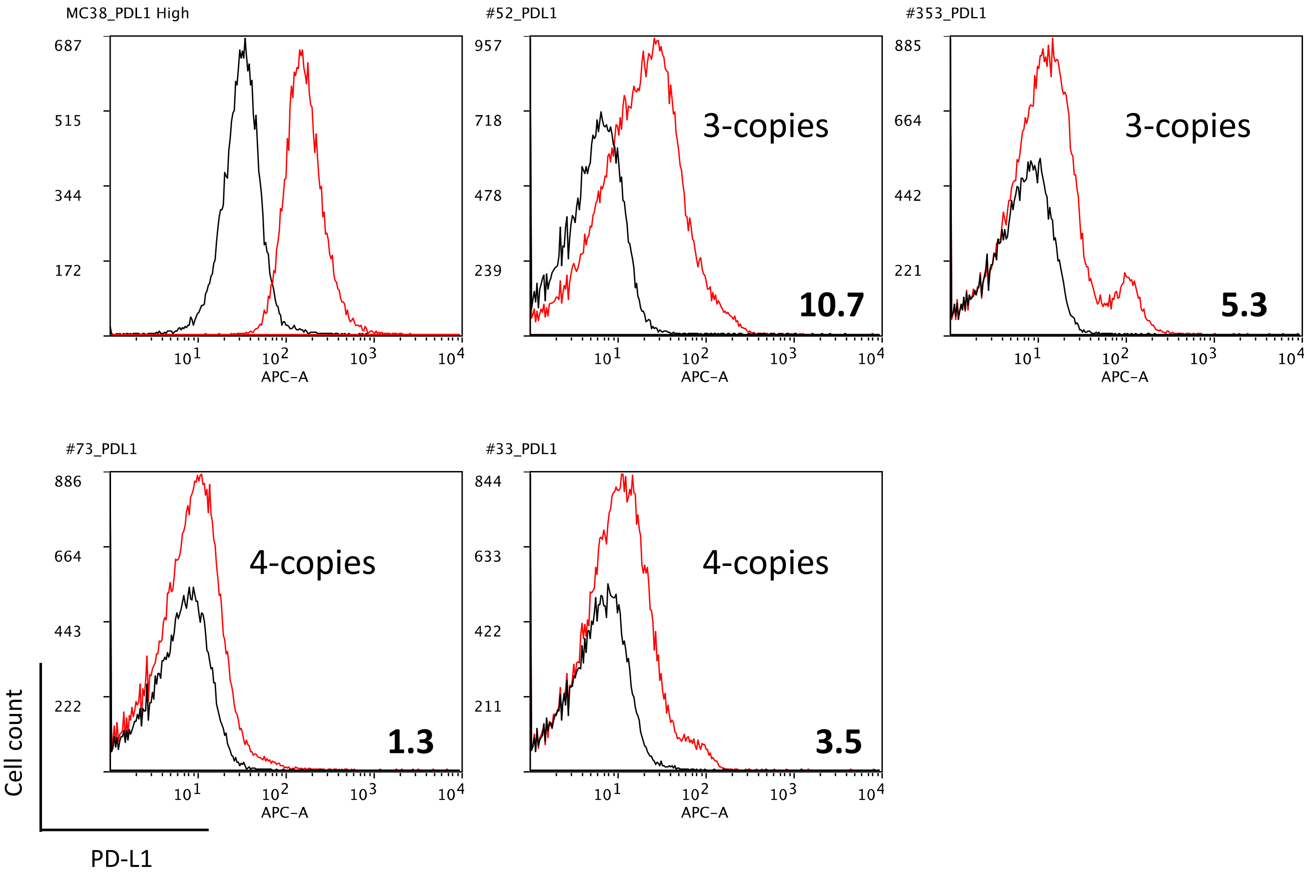

Supplementary Figure 3. FACS analysis of PD-L1 expression in E***μ***-*Myc* lymphomas shown to have varying *Cd274* (PD-L1) genomic copy-number. MC38 is a murine colorectal carcinoma cell line and PD-L1(Hi) control. #52 and #353 harbour 3-copies, #73 and #33 harbour 4 copies. Viable cells were gated based on morphology and CD220 positivity (not shown). Rat IgG2α isotype control (black), PD-L1 streptavidin APC - biotin APC Cy7 (red), difference in geometric mean fluorescence intensity for PDL1 fluorochrome compared to isotype control (bold text).

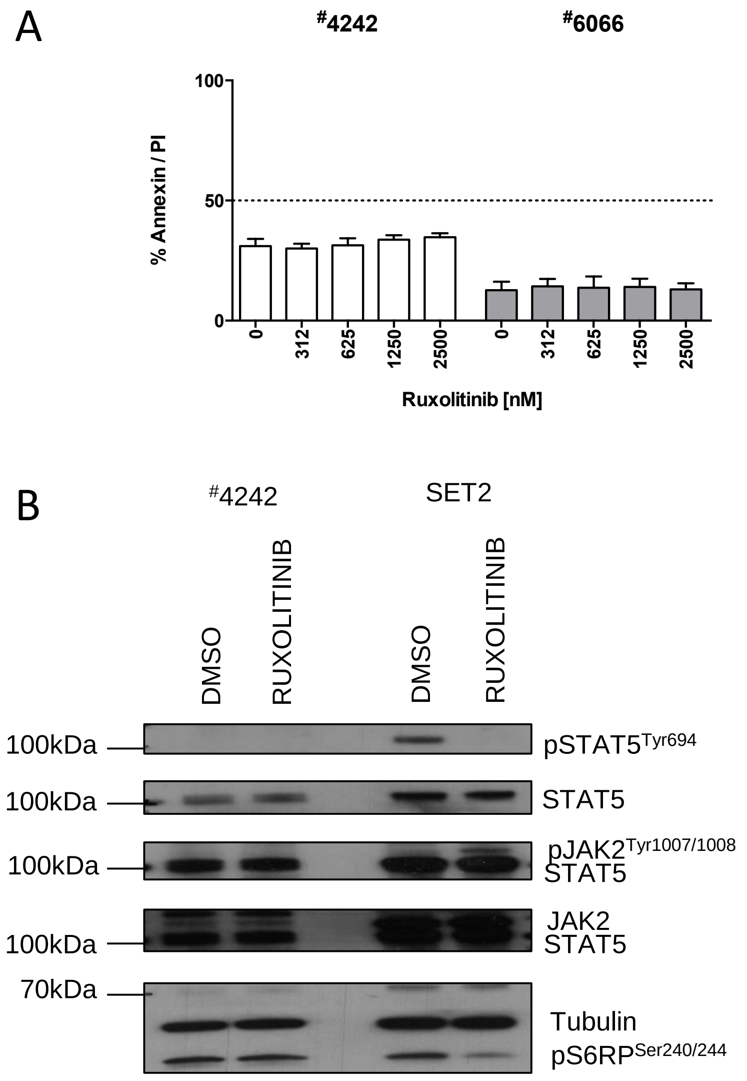

Supplementary Figure 4. JAK2 inhibition with ruxolitinib is insufficient to effect apoptosis of E***μ***-*Myc* lymphoma. A) E*μ-Myc* lymphomas #4242 and #6066 (left) were cultured in the presence of doubling dilutions of ruxolitinib for 24 hours prior to flow cytometric analysis for annexin-V / propidium iodide uptake. Human SET2(V617F) cell line (right) was used as positive control for ruxolitinib, cultured in RPMI media with doubling dilutions of ruxolitinib for 48 hours prior to flow cytometric analysis for annexin-V / propidium iodide uptake. Data shown is mean annexin-V/PI positive population ± SEM for triplicate experiments. B) E*μ-Myc* lymphoma #4242 and human SET2(V617F) cell lines were exposed to 1uM ruxolitinib or DMSO vehicle control for three hours prior to protein extraction and separation with SDS-PAGE. Immunoblotting was performed to show expression of pSTAT5, STAT5, pJAK2, JAK2, pS6RP and tubulin loading control.

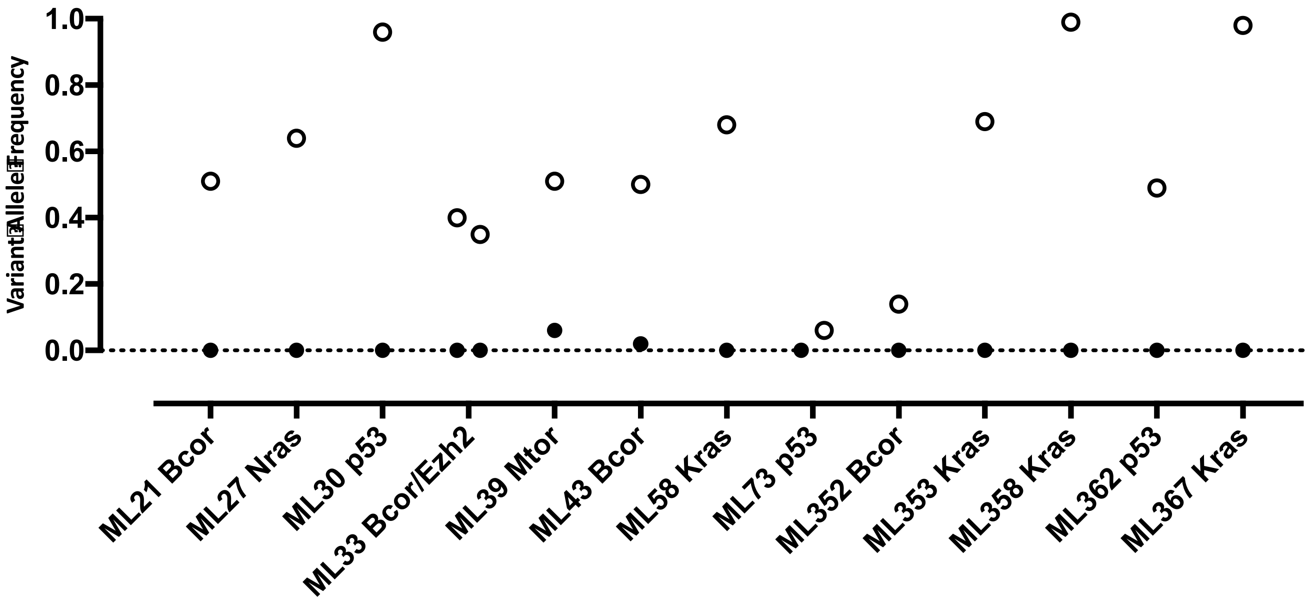

Supplementary Figure 5. Variant allele frequency determined by targeted amplicon sequencing. Targeted amplicon sequencing (TAM-seq) was performed on DNA extracted from peripheral blood at 4-weeks of age (filled circles) and at end-stage (empty-circles) to validate somatic mutations in E*μ-Myc* lymphomas. Candidate driver mutations were either undetectable or present at very low frequencies (<0.1) at 4-weeks of age and in each case were found at increased levels at end-point.

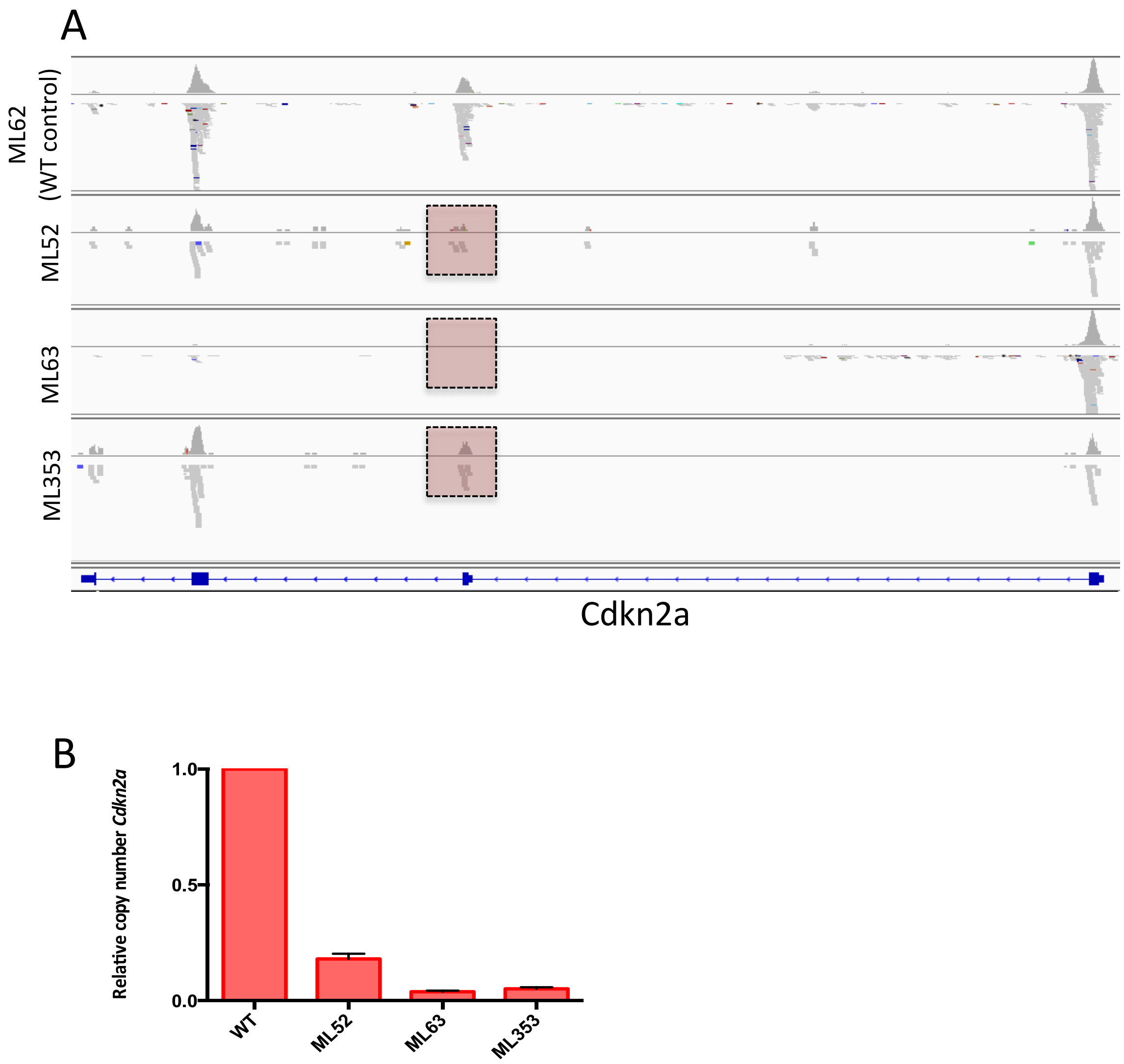

Supplementary Figure 6. Identification of focal deletions in cdkn2a. A) Whole exome sequencing analysis of read-pile up across the *Cdkn2a* locus showing potential *Cdkn2a* loss in ML52, ML63 and ML535 compared with WT littermate control (ML62). Dotted boxes indicate areas of reduced read depth indicative of copy number loss B) QRT-PCR displaying relative copy number for *Cdkn2a* confirms *Cdkn2a* deletion in ML52, ML63 and ML353 compared to WT littermate control (ML62). Data shown is mean relative copy number ± SEM, three replicates.

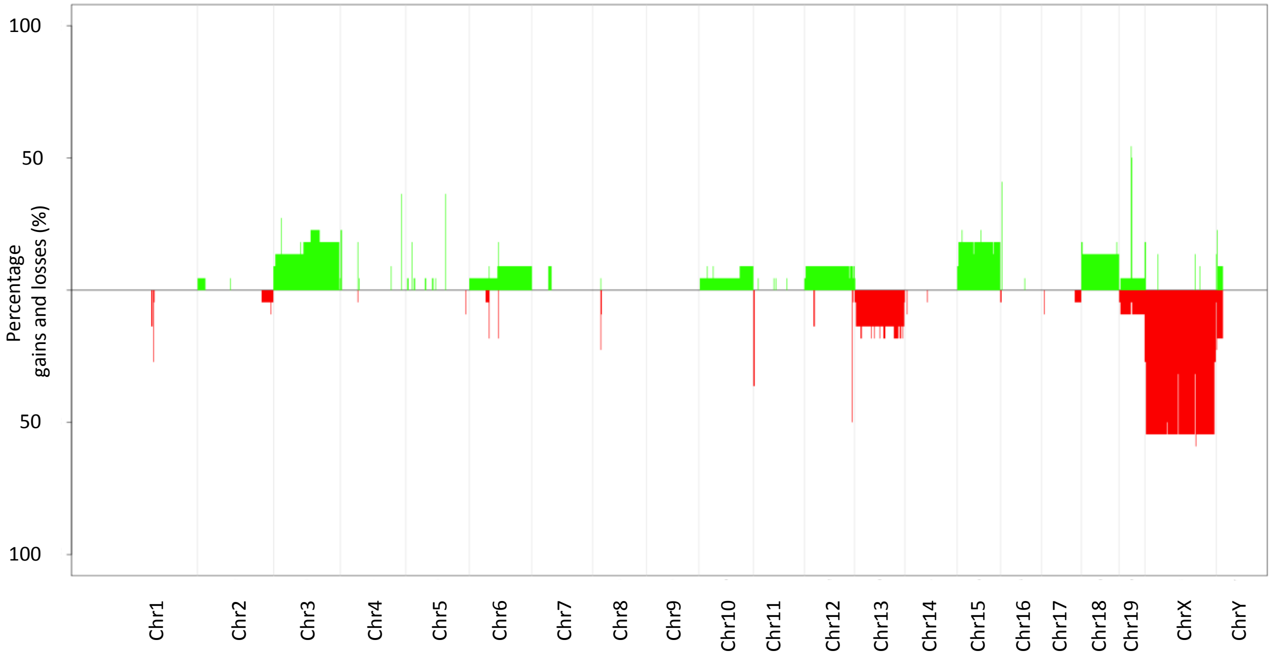

Supplementary Figure 7. Somatic copy-number alterations detected in Eμ-*Myc* lymphomas by whole-exome or low to medium coverage whole-genome sequencing. Overlapping regions of gain (green) or loss (red), are shown as a percentage of cases (22 cases analyzed) and are plotted against genome position (chromosome number).

A

Well	Cell line	Barcode
A1	4242	GTCTGAATGAAGTCGA
A4	4242	CTCTGTGCGAGGAATT
C2	4242	CTAGCTGTCTGCTGCG
D2	6066	CAATCAGTGAGGTAGA
D11	6066	CACTGTATCAAAGATA
F8	6066	GTCGCTATGACACATA

B

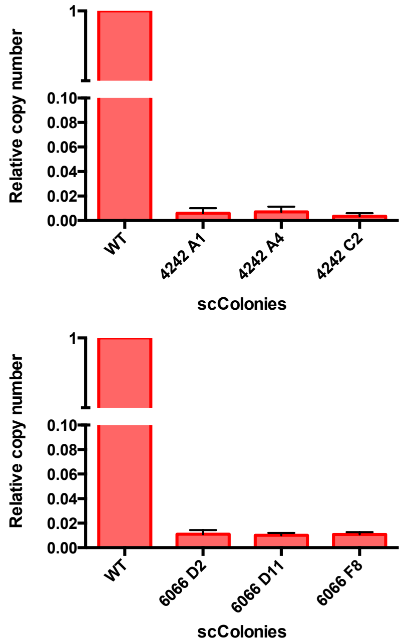

C

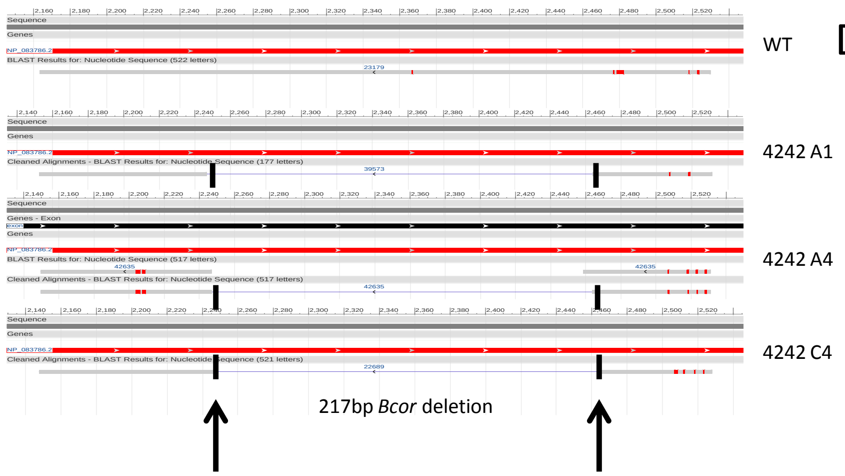

D

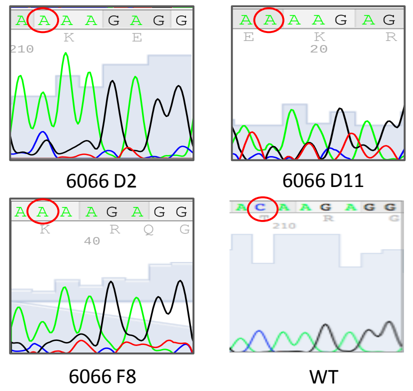

Supplementary Figure 8. Validation of coalescing driver mutations in #4242 and #6066 E***μ***-*Myc* clones. Cell lines were retrovirally transduced with an inert DNA ‘barcode’ and BFP fluorescent reporter at an MOI of 0.1. The cells were single-cell sorted on BFP expression and cultured to generate single-cell-derived colonies (scColonies). A) scColonies were sanger sequenced to show the presence of one barcode. B) QRT-PCR was performed to show total lack of *Cdkn2a* copies relative to WT control. Data shown is mean relative copy number ± SEM, three replicates. C) Sanger sequencing was performed on the 4242 scColonies and a BLAST readout highlights the 217bp deletion in *Bcor* apparent in the 4242 compared to WT. D) Sanger sequencing across *Nras* confirms C-to-A mutation corresponding to Q61K point mutation in the 6066 scColonies compared to WT

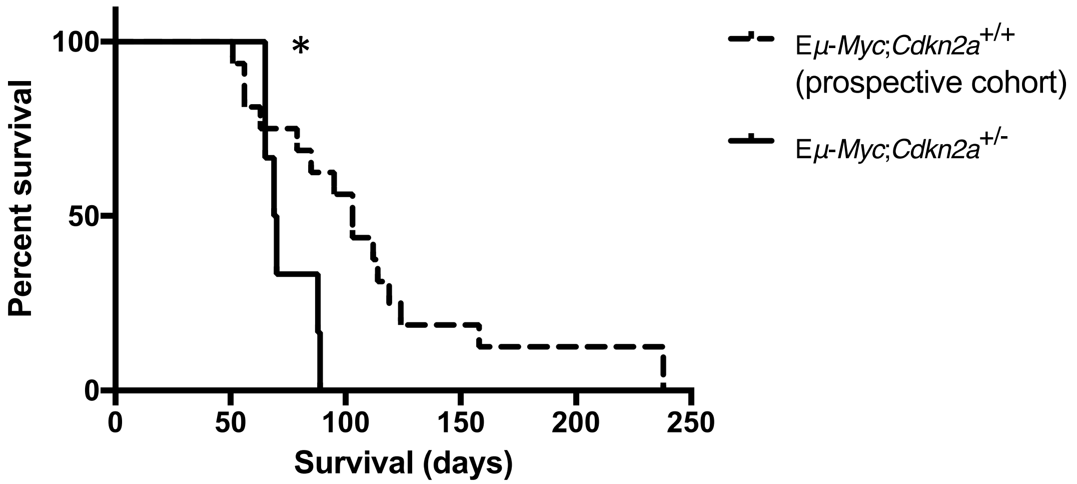

Supplementary Figure 9. E***μ***-*Myc*;*Cdkn2a*$^{+/-}$ mice have significantly accelerated mortality. Kaplan-Meier curve showing transgenic E*μ*-*Myc*;*Cdkn2a*$^{+/-}$ mice (solid line, n=6) and E*μ*-*Myc*;*Cdkn2a*$^{+/+}$ transgenic mice (dashed line, n=16), with a median survival time of 69.5 days and 103 days, respectively. * = p value < 0.05 log-rank (mantel-cox) test.

A

GENE	CHR	POS	REF	ALT	CONSEQUENCE	REF DEPTH	ALT DEPTH	VAF
Kras	6	123124	T	G	Q61H	58	37	.67

B

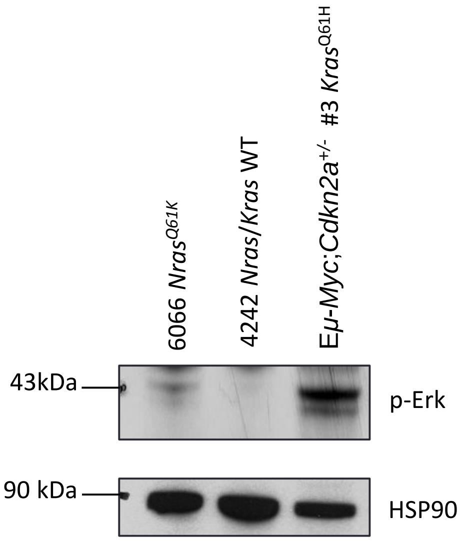

C

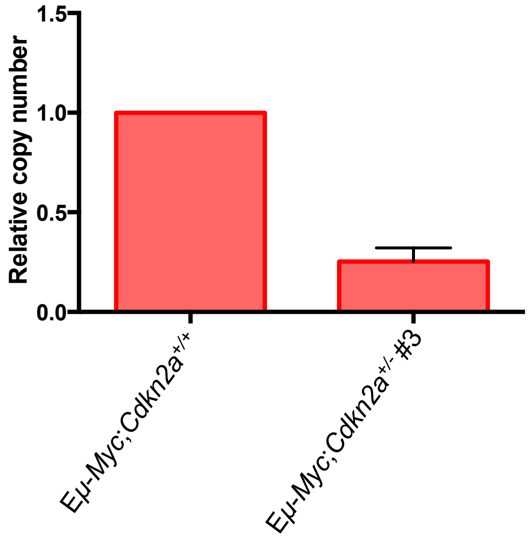

Supplementary Figure 10. Secondary and tertiary somatic mutations in E**μ**-*Myc*;*Cdkn2a*$^{+/-}$ lymphomas. A) Whole exome-sequencing readout showing the somatically acquired activating *Kras* mutation found in 1/6 E*μ*-*Myc*;*Cdkn2a*$^{+/-}$ lymphomas. B) Western blot analysis showing elevated phosphorylated ERK (p-ERK) in *Nras* mutant 6066 and E*μ*-*Myc*;*Cdkn2a*$^{+/-}$ #3 lymphoma indicating activated RAS MAPK pathway signaling. C) QRT-PCR analysis of the *Cdkn2a* locus in lymphoma E*μ*-*Myc*;*Cdkn2a*$^{+/-}$ #3 showing somatic deletion compared to E*μ*-*Myc*;*Cdkn2a*$^{+/+}$. Data shown is mean relative copy number ± SEM, three replicates.

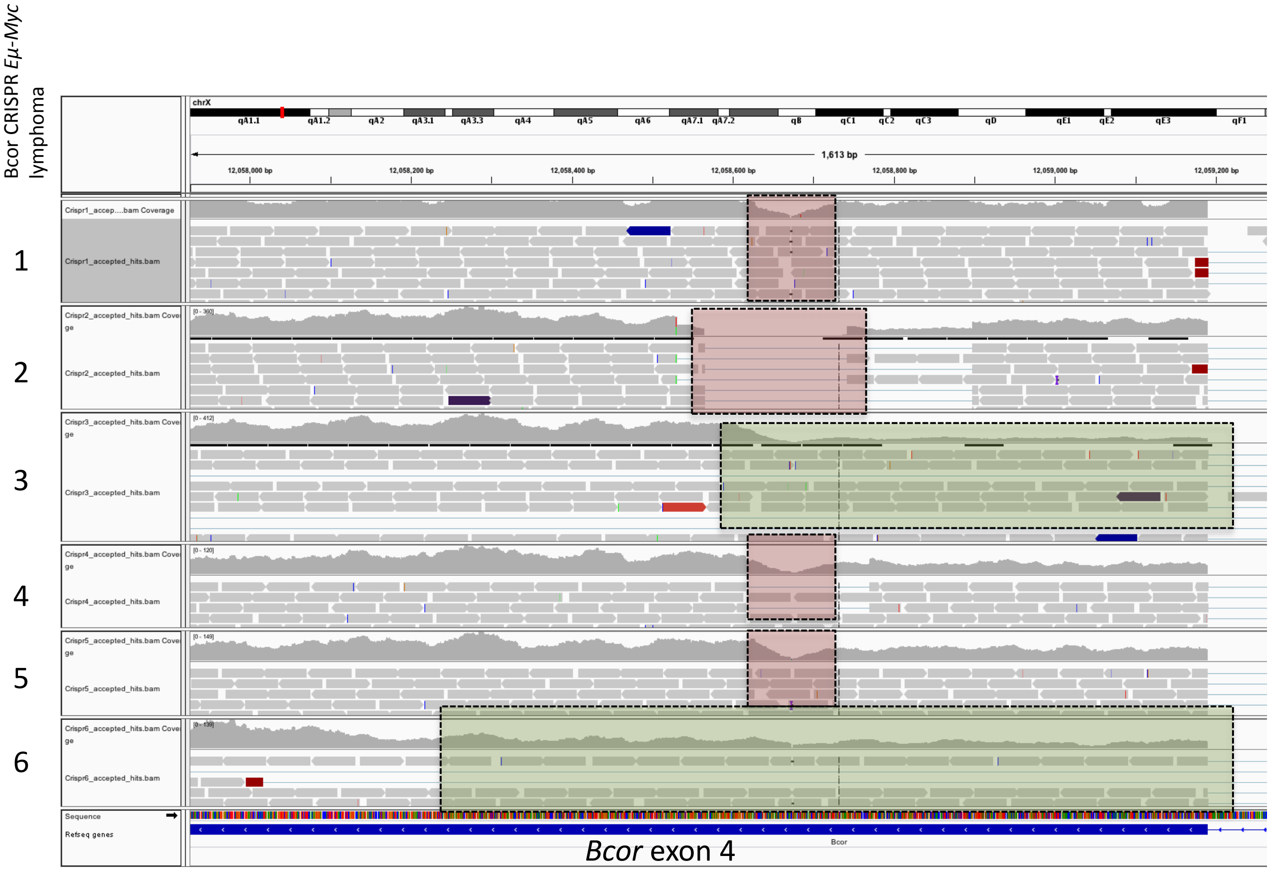

Supplementary Figure 11. RNA-seq IGV browser view showing CRISPR-Cas9 mediated *Bcor* insertions and deletions in E***μ***-*Myc* lymphomas. Red boxes represent small deletion events and green boxes represent reduced read depth, likely due to a large deletion event.

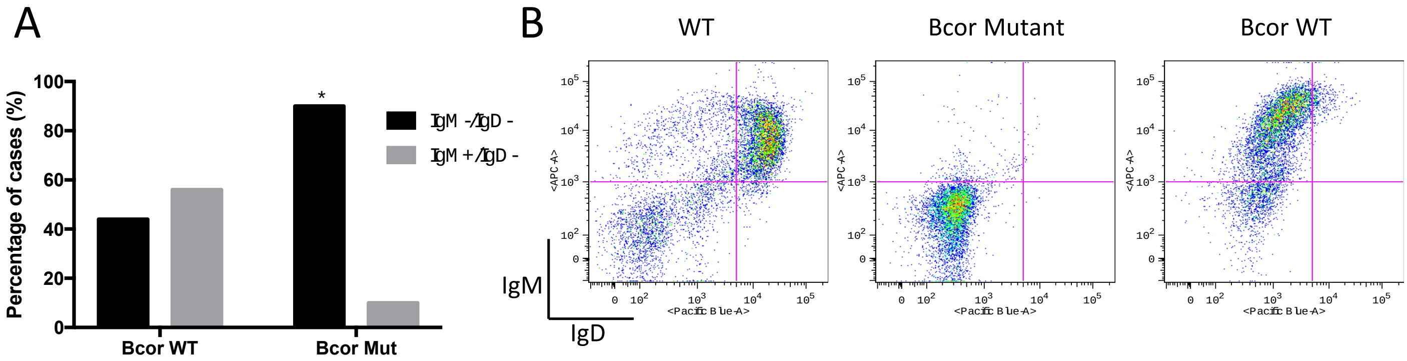

Supplementary Figure 12. Surface immunoglobulin profile of B-cells in *Bcor* mutant and *Bcor* WT sporadic and fetal liver cell-derived E***μ***-*Myc* tumours. A) Surface IgM and IgD were assessed in the prospective E*μ*-*Myc* cohort (four *Bcor* mutant tumours and 12 *Bcor* WT tumours) and 12 fetal liver derived E*μ*-*Myc* tumours (six *Bcor* mutant tumours (*Bcor* KD) and six *Bcor* WT tumours (p53 KD)). 90% of *Bcor* mutant tumours were IgM-/IgD- while 10% were IgM+/IgD-. 44.4% pf *Bcor* WT tumours were IgM-/IgD- while 56.6% were IgM+/IgD-. *Bcor* mutations are associated with an IgM-/IgD- profile (Chi-squared test, p<0.05). B) Representative IgM/IgD FACS plots showing WT bone marrow B-cells, Bcor mutant E*μ*-*Myc* tumour cells and *Bcor* WT E*μ*-*Myc* tumour cells.

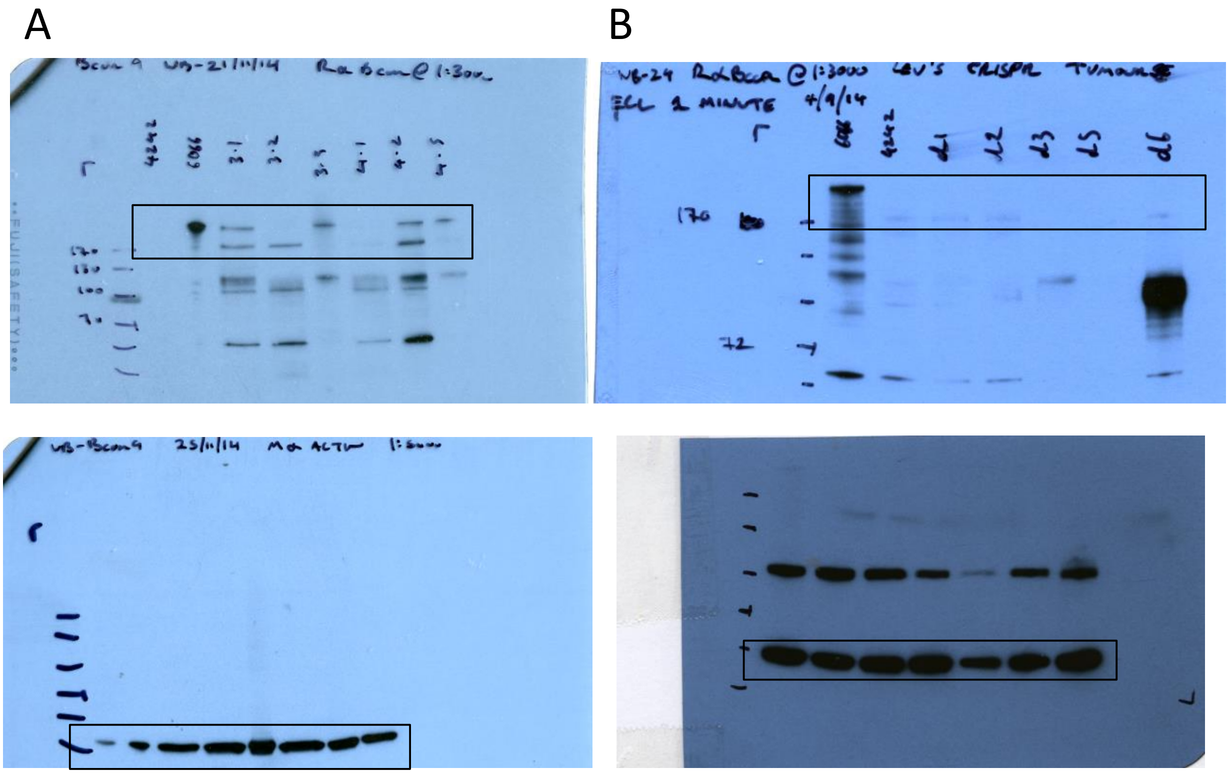

Supplementary Figure 13. Unedited western blots shown in main Figure 4. Black boxes correlate with the edited images shown in the main text. A) Original blots shown in Figure 4D and B) Original blots shown in Figure 4E.

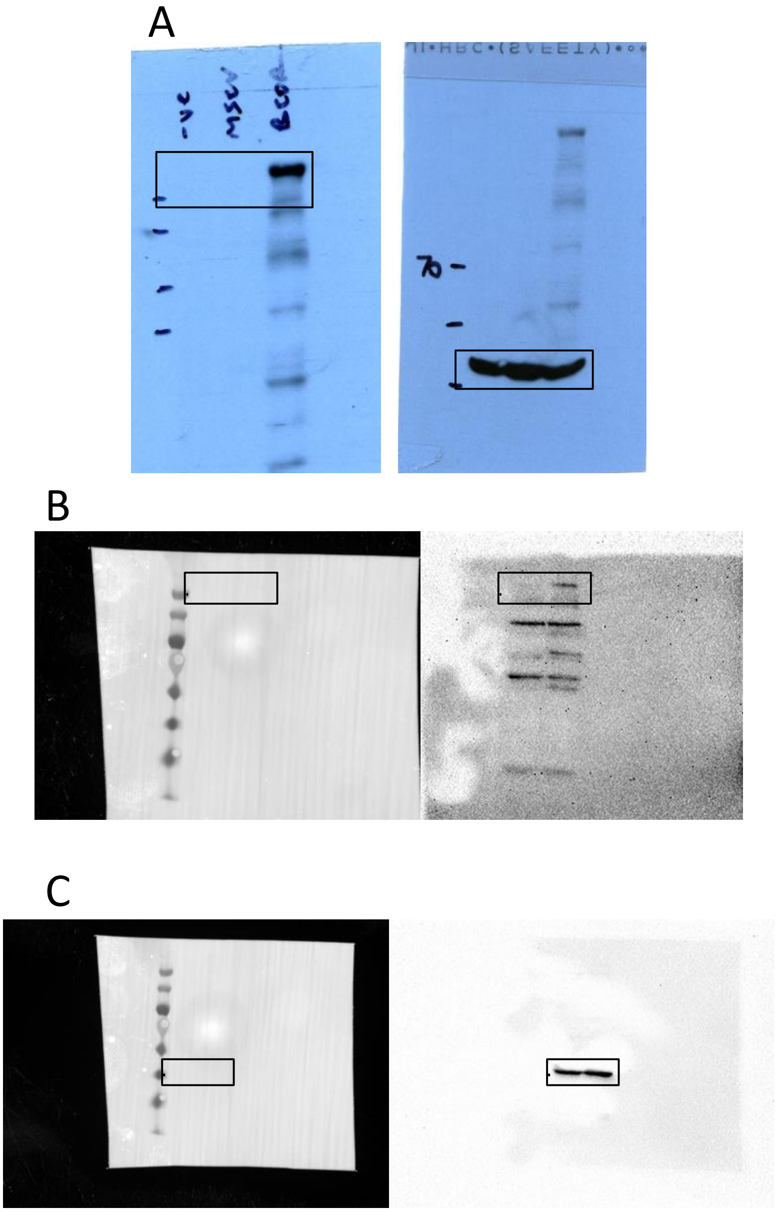

Supplementary Figure 14. Unedited western blots shown in main Figure 5. Black boxes correlate with the edited images shown in the main text. A) Original blots shown in Figure 5B, B) Original blots shown in Figure 5D upper-panel and C) original blots shown in Figure 5D lower-panel. For B) and C) western blots were imaged using the BioRad Chemidoc, which provides colorimetric images (protein ladder) and chemiluminescent images (protein bands) from which a composite image can be made giving molecular weight estimates against protein bands.

Supplementary Table 1. Summary of Eu-Myc mice and disease presentation

ID	Sex	Latency (days)	Generation	Relation ship	IgM/IgD profiles	WBC (10^9 cells/L)	Presentation
ML20	F	124	Prospective B	Father 371	IgM lo IgD- / IgM hi IgD-	108.2	Splenic/thymic
ML21	F	119	Prospective B	Father 371	IgM- IgD-	34.2	Splenic/Nodal
ML27	F	51	Prospective B	Father 371	IgM lo IgD-	121.6	Splenic/Nodal
ML30	F	95	Prospective B	Father 371	IgM- IgD-	49.4	Splenic/Nodal
ML33	F	103	Prospective B	Father 371	IgM- IgD-	64.8	Splenic/Nodal
ML39	F	168	Prospective B	Father 371	IgM- IgD-	45.2	Splenic/Nodal
ML43	F	79	Prospective B	Father 371	IgM- IgD-	50.4	Splenic/Nodal
ML52	F	85	Prospective B	Father 371	IgM hi IgD-	461.4	Thymic/Nodal
ML58	M	63	Prospective B	Father 371	IgM- IgD-	158.8	Splenic/Nodal
ML63	M	103	Prospective B	Father 371	IgM hi IgD-	94	Splenic/Nodal
ML73	F	114	Prospective B	Father 371	IgM lo IgD- / IgM hi IgD-	145.2	Splenic/Thymic
ML352	F	130	Prospective A	Father 288	IgM- IgD-	5	Splenic/Nodal
ML353	F	238	Prospective A	Father 288	IgM lo IgD- / IgM hi IgD-	36.8	Splenic/Nodal
ML358	M	56	Prospective A	Father 288	IgM- IgD-	42.2	Splenic/Nodal
ML362	F	56	Prospective A	Father 288	IgM hi IgD-	65.8	Splenic/Nodal
ML367	M	158	Prospective A	Father 288	IgM- IgD-	94	Splenic/Nodal
#4242 (cell line)	M	N/A	Retrospective	none	n/a	n/a	n/a
#6066 (cell line)	M	N/A	Retrospective	none	n/a	n/a	n/a
#299	M	126	Retrospective	none	n/a	n/a	n/a
#88	M	N/A	Retrospective	none	n/a	n/a	n/a
#13	M	189	Retrospective	none	n/a	n/a	n/a
#218	M	218	Retrospective	Brother 219	n/a	n/a	n/a
#219	M	105	Retrospective	Brother 218	n/a	n/a	n/a

Supplementary Table 2. shRNA and CRISPR-Cas9 sequences

**Gene ID**	**Hairpin ID**	**Hairpin Sequence**
Bcor	Bcor9	TGCTGTTGACAGTGAGCGACGAGAAGAAGCAGACACTAAATAGTGAAGCCACAGATGTATTTAGTGTCTGCTTCTTCTCGG TGCCTACTGCCTCGGA
trp53	p53.1224	CTCGAGAAGGTATATTGCTGTTGACAGTGAGCGCCCACTACAAGTACATGTGTAATAGTGAAGCCACAGATGTATTACACA TGTACTTGTAGTGGATGCCTACTGCCTCGGAATTC
n/a	scrambled	TGCTGTTGACAGTGAGCGATCTCGCTTGGGCGAGAGTAAGTAGTGAAGCCACAGATGTACTTACTCTCGCCCAAGCGAGA GTGCCTACTGCCTCGGA
**Gene ID**	**crispr trigger ID**	**crispr trigger sequence**
Bcor	BcorG2	CAGTGGCTGGGCCAAGCCGT
Trp53	p53(b)	GAAGTCACAGCACATGACGG
